# The Association between Apolipoprotein B, Cardiovascular Risk Factors and Subclinical Atherosclerosis—Findings from the SEPHAR National Registry on Hypertension in Romania

**DOI:** 10.3390/ijms24032813

**Published:** 2023-02-01

**Authors:** Maria Dorobanțu, Vasile-Bogdan Halațiu, Oana Gheorghe-Fronea, Cornelia-Gabriela Bala, Horațiu Moldovan, Raluca Irinel-Parepa, Ioana-Patricia Rodean, Imre Benedek, Theodora Benedek

**Affiliations:** 1 Faculty of Medicine, University of Medicine and Pharmacy “Carol Davila”, 020021 Bucuresti, Romania; 2 Faculty of Medicine, “George Emil Palade” University of Medicine, Pharmacy, Science and Technology, 540139 Târgu Mures, Romania; 3 Cardiology Department, Emergency Clinical County Hospital, 540136 Târgu Mures, Romania; 4 Cardiology Department, Emergency Clinical Hospital Bucharest, 014461 Bucharest, Romania; 5 Faculty of Medicine, “Iuliu Hațieganu” University of Medicine and Pharmacy, 400347 Cluj-Napoca, Romania; 6 Department of Cardiovascular Surgery, Bucharest Clinical Emergency Hospital, 014461 Bucharest, Romania; 7 Faculty of Medicine, “Ovidius” State University Constanta, 900470 Constanta, Romania

**Keywords:** apolipoprotein B, atherosclerosis, carotid stenosis, lipid profile, serum uric acid

## Abstract

The present study aimed to investigate the association between apolipoprotein B (Apo B) and classical features associated with clinical or subclinical atherosclerosis. A total of 811 adult patients from the general Romanian population, included in the national SEPHAR registry on hypertension, were divided into two groups based on Apo B value (low versus high Apo B with a cut-off established at 130 mg/dL) and subsequently into four subgroups according to the cut-offs recommended by the 2021 ESC Guidelines on Cardiovascular Disease Prevention. In all patients, lipid profile, uric acid, full blood count and presence of significant carotid plaques were assessed. Apo B levels were positively correlated with proatherogenic lipids (total cholesterol, triglycerides and LDL-cholesterol, *p* < 0.0001) and negatively correlated with HDL cholesterol (all *p* < 0.05). In comparison with patients with low Apo B levels, those with elevated Apo B levels more frequently presented significant carotid plaques (17% vs. 19% vs. 28% vs. 46%, *p* < 0.0001). Univariate regression analysis identified a strong association between the level of uric acid and increased value of Apo B in the four subgroups (uric acid 4.8 +/− 1.3 vs. 5 +/− 1.6 vs. 5.1 +/− 1.5 vs. 5.8 +/− 1.6, r = 0.2, *p* < 0.0001). The results of this nationwide registry on hypertension in Romania indicate that high Apo B may be considered as a risk factor for CVD, promoting atherosclerosis and associated with increased expression of classical markers of clinical or subclinical CVD.

## 1. Introduction

Cardiovascular disease (CVD) remains the main cause of morbidity and mortality, accounting for almost one third of deaths worldwide [1,2]. In Romania, CVD constitutes more than 62% of the causes of death [3]. The continuous increase in the prevalence of cardiovascular risk factors predicts a bleak future, with recent trends indicating an increase in cardiovascular mortality rates [4]. 

The vast majority of CVDs are associated with atherosclerosis. Atherosclerosis is a process initiated early in life and remains asymptomatic for a long time. Therefore, the early detection of atherosclerotic disease before the onset of symptoms represents an important goal in modern cardiology [5,6]. In the pathogenesis of atherosclerosis, both atherogenic lipids and inflammation play a central role, contributing not only to the initiation but also to the progression of atherosclerotic plaques [7]. Atheromatous plaque instability may occur at any time during atherosclerosis progression and is the main contributor to acute atherosclerosis-related complications, such as acute coronary syndromes [8,9]. 

**Figure 1:**
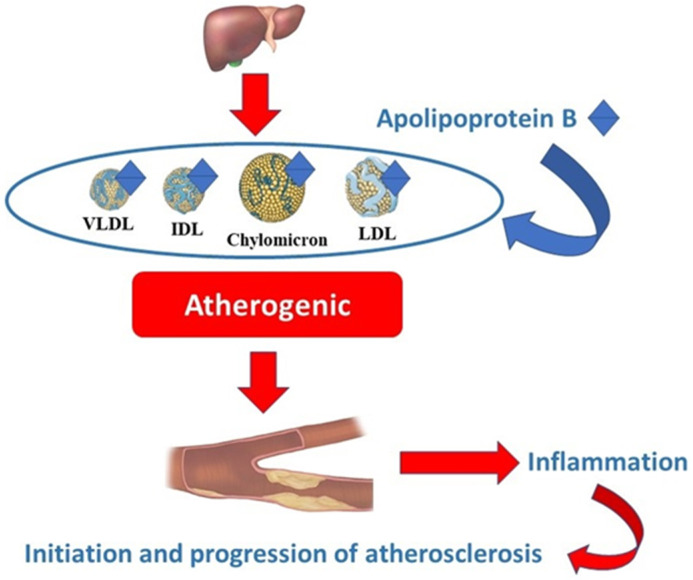
The link between Apo B and atherosclerosis. VLDL—very low-density lipoprotein; IDL—intermediate-density lipoprotein; LDL—low-density lipoprotein.

Among lipids, low-density lipoproteins (LDL) are the most important in atherogenesis, having a primary role in cholesterol transport [10]. The modulation of the lipid profile has become one of the most relevant goals for cardiovascular prevention, mostly reducing cardiovascular risk by targeting LDL-cholesterol with different lowering molecules. In recent times, attention has also been directed toward compounds that better reflect pro-atherogenic risk. Lately, an important role in atherosclerosis has been attributed to apolipoproteins, which are involved in regulating lipoprotein metabolism [11]. Of the different types of apolipoprotein, apolipoprotein B (Apo B) is an essential component of all atherogenic lipids, such as very low-density lipoproteins (VLDLs), intermediate density lipoproteins (IDLs), LDLs and chylomicrons [12]. According to the literature, there is significant variability in the lipid composition of Apo B lipoproteins. At present, Apo B is considered superior to total cholesterol and triglyceride levels for predicting cardiovascular risk [13]. The link between Apo B and atherosclerosis is illustrated in Figure 1. Data regarding the predictive role of Apo B versus LDL-cholesterol are controversial. A series of studies have shown that Apo B is a more accurate predictor of cardiovascular risk than LDL-cholesterol and non-high-density lipoprotein cholesterol (non-HDL) [13,14]. On the other hand, a recent study that included over 300,000 patients did not demonstrate the superiority of Apo B over LDL-cholesterol for the assessment of cardiovascular risk [15]. The same information is included in the 2021 ESC Guidelines on Cardiovascular Disease Prevention in Clinical Practice [16]. Thus, a series of studies is needed to establish the role of Apo B as a predictor of CVDs.

Therefore, the current study aimed to investigate the association between Apo B and classical features associated with clinical or subclinical atherosclerosis.

## 2. Results

### 2.1. General Characteristics of the Study Populations in the Low versus High Apo B Groups

Patients with an Apo B level above 130 mg/dL were significantly older than those with levels below 130 mg/dL (57.6 ± 11.9 vs. 50.4 ± 16.8; *p* < 0.0001) and were more frequently male (45% vs. 31% *p* = 0.008). No significant difference was observed between the two groups in terms of family history of premature CVD (32% vs. 27%; *p* > 0.05) or personal history of premature CVD (11% vs. 9%; *p* > 0.05). The differences between the two groups in terms of atherosclerosis risk factors and the presence of cardiovascular and noncardiovascular comorbidities are presented in Table 1. 

**Figure 2:**
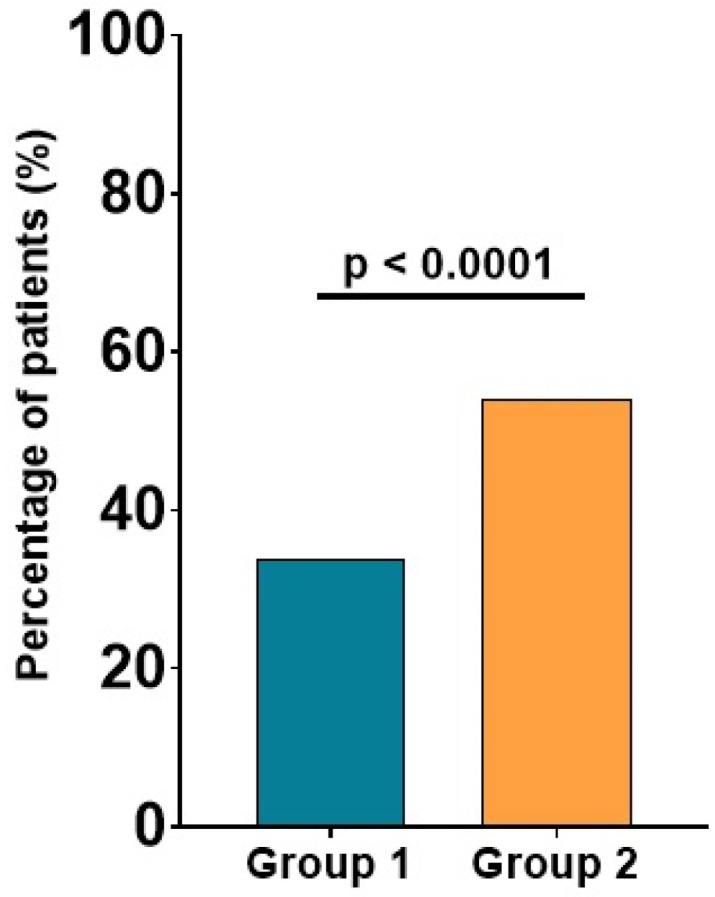
Significant carotid plaques in patients with low versus high Apo B. The values are expressed as percentages; *p*-values refer to between-group comparisons based on a chi-squared test.

In comparison with patients with low Apo B levels, those with elevated Apo B levels more frequently presented significant carotid plaques, evidenced by carotid ultrasound (54% vs. 34%, *p* < 0.0001), as shown in Figure 2.

As expected, patients with increased values of Apo B presented significantly higher values of serum lipids (total cholesterol, LDL-cholesterol and triglycerides) and significantly lower values of HDL-cholesterol, as shown in Table 2. Biochemistry also revealed significantly higher values of uric acid (5.2 ± 1.5 mg/dL vs. 6.2 ± 1.7 mg/dL; *p* = 0.001), fasting glucose (99.0 ± 20.9 mg/dL vs. 110.4 ± 37.0 mg/dL; *p* < 0.0001) and glycosylated hemoglobin (5.6 ± 0.7% vs. 5.9 ± 1.1%; *p* < 0.0001) in patients with Apo B values above 130 mg/dL.

### 2.2. Characteristics of the Study Populations in the Four Subgroups According to Apo B Level

**Figure 3:**
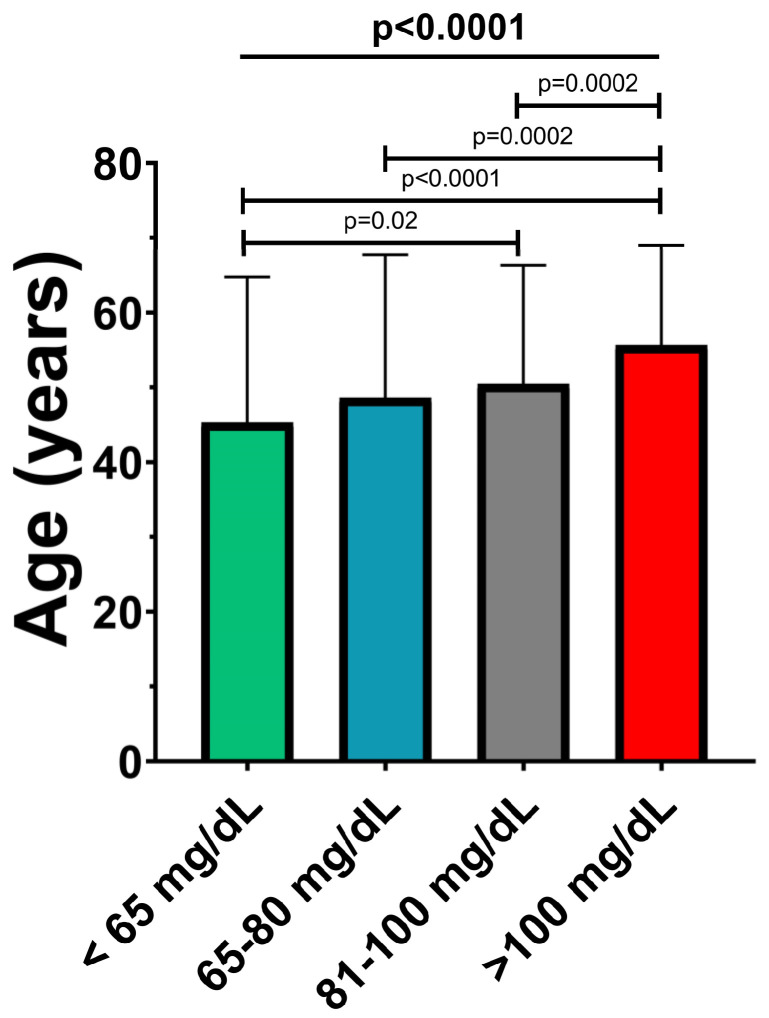
Mean age of patients in the Apo B risk subgroups. The values are expressed as mean ± standard deviation; *p*-values refer to between-group comparisons based on ANOVA or Mann–Whitney tests.

As shown in Figure 3, a direct association was observed between Apo B and age, with patients being significantly older in the higher risk groups (44.8 ± 19.2 vs. 48.2 ± 19.1 vs. 50.1 ± 15.8 vs. 55.5 ± 13.2; *p* < 0.0001).

**Figure 4:**
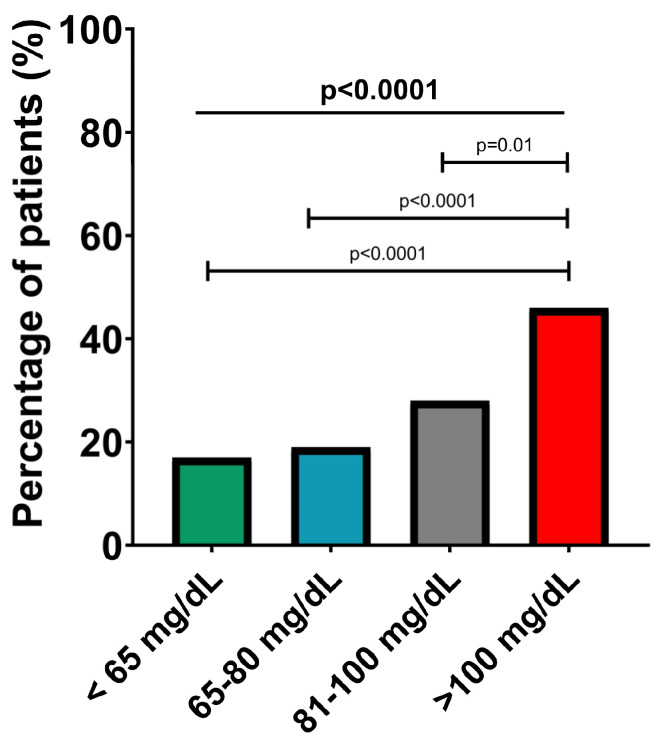
Presence of significant carotid plaques in the Apo B risk subgroups. The values are expressed as percentages; *p*-values refer to between-group comparisons based on a chi-squared test.

A significant progressive increase in the percentage of patients with carotid plaques was also observed, from the subgroup with Apo B below 65 md/dL to the group with Apo B above 100 mg/dL (17% vs. 19% vs. 28% vs. 46%, *p* < 0.0001), as shown in Figure 4. No other differences between the four groups were found in terms of atherosclerosis risk factors (smoking, obesity, hypertension and diabetes mellitus) or the presence of cardiovascular and non-cardiovascular comorbidities (all *p* > 0.05).

Lipid profile analysis in the four subgroups identified a statistically significant progressive increase in total cholesterol, LDL-cholesterol and triglycerides from group A to group D, in parallel with a significant progressive decrease of HDL-cholesterol, as shown in Table 3.

At the same time, a significant increase in uric acid, fasting glucose level and glycosylated hemoglobin were observed, as indicated in Table 4.

**Figure 5:**
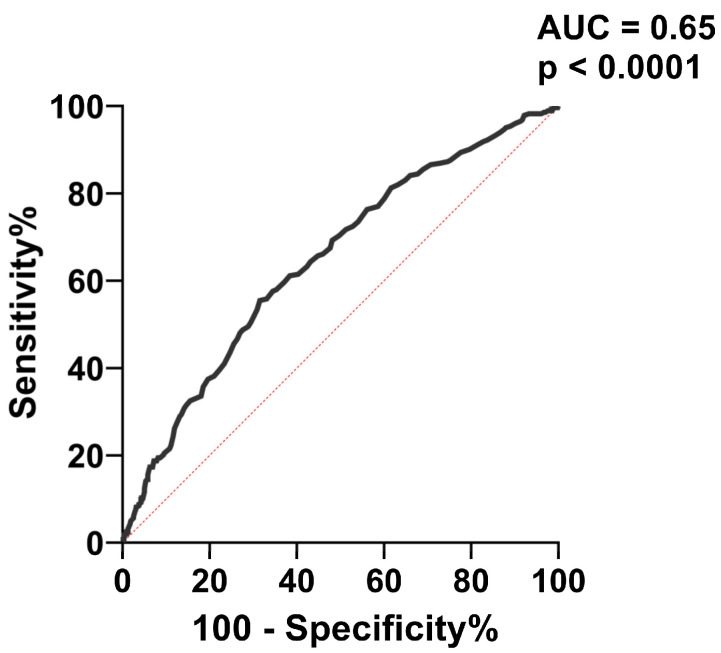
ROC analysis for the accuracy of Apo B level in predicting carotid plaques. AUC = area under the curve.

To further evaluate the capacity of Apo B to predict carotid plaques, we performed receiver operating characteristic (ROC) curve analysis (Figure 5). The best cut-off value of Apo B for predicting carotid plaques was > 0.955 mg/dL, with associated sensitivity (61.13%) and specificity (61.60%).

### 2.3. Apolipoprotein B, Uric Acid and Serum Lipids

**Figure 6:**
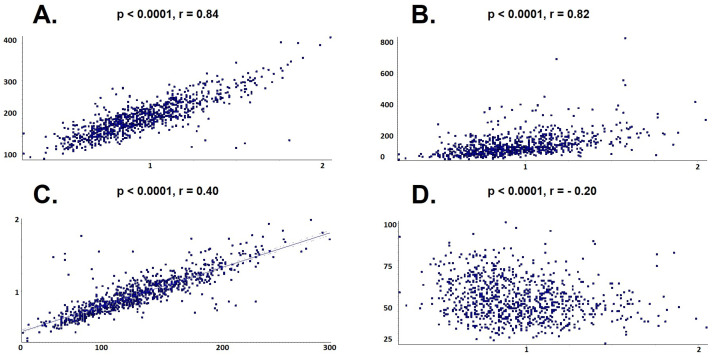
Univariate linear regression between Apo B and lipid profile. (**A**) Total cholesterol; (**B**) Triglycerides; (**C**) Low-density lipoprotein cholesterol; (**D**) High-density lipoprotein cholesterol.

Univariate regression analysis showed a direct and strong relationship between Apo B and serum levels of total cholesterol (*p* < 0.0001, r = 0.84), LDL-cholesterol (*p* < 0.0001, r = 0.82) and triglycerides (*p* < 0.0001, r = 0.4), in parallel with an inverse proportional relationship between Apo B and HDL-cholesterol level (*p* < 0.0001, r = −0.2), as shown in Figure 6.

**Figure 7:**
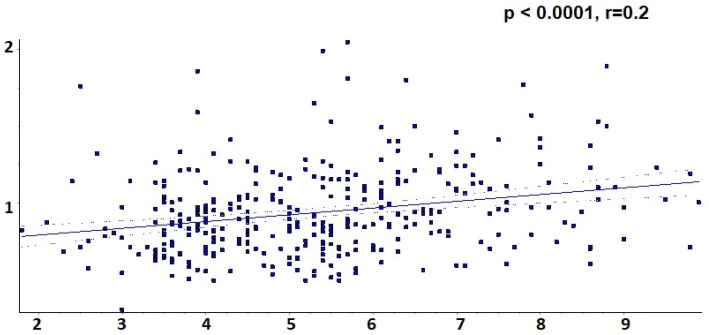
Univariate linear regression between Apo B and uric acid.

At the same time, univariate regression analysis identified a strong association between the level of uric acid and increased Apo B value, as indicated in Figure 7 (uric acid 4.8 +/− 1.3 in subgroup A, 5.0 +/− 1.6 in subgroup B, 5.1 +/− 1.5 in subgroup C and 5.8 +/− 1.6 in subgroup D, r = 0.2, *p* < 0.0001).

When adding the presence of carotid plaque, creatinine serum level and HbA1c to the multiple logistic regression analysis, the power of the lipid profile and uric acid serum level for predicting Apo B level increased to 84.51% (*p* < 0.0001).

## 3. Discussion

In this study, we report the results of a subanalysis of the first nationwide registry on hypertension in Romania—the SEPHAR study. Our study is focused on the presence of elevated Apo B in the population of the SEPHAR registry and indicates that high Apo B may be considered as a risk factor for CVD, being associated with clinical or subclinical markers of atherosclerosis.

### 3.1. Apolipoprotein B and Lipid Profile

In the current study, we observed a positive correlation between Apo B levels and proatherogenic lipids (total cholesterol, triglycerides and LDL-cholesterol) and a negative correlation with HDL-cholesterol. The process by which Apo B is involved in atherogenesis is complex. Apo B capture in the arterial wall is the main initiating factor in atherogenesis. This leads to the accumulation of lipids, triggering a cellular response in the arterial wall, which results in the acceleration of the lipid accumulation process via secretory sphingomyelinase, lipoprotein lipase and phospholipase A2 enzymes [17]. In a population of over 300,000 patients without a history of cardiovascular disease or lipid-lowering treatment, Apo B was highly correlated with total cholesterol, LDL-cholesterol and non-HDL-cholesterol (all *p* < 0.001; all r > 0.9) [15]. Cho et al. reported an association between Apo B and total cholesterol, LDL-cholesterol and triglycerides but not with HDL-cholesterol in an Asian cohort [18]. Moreover, they developed a model for predicting Apo B value using LDL-cholesterol and triglycerides. The level of concordance between Apo B and LDL-cholesterol varied between 47% and 56% in a population of over 11,000 patients in whom the correlation was observed between Apo B and LDL-chol and non-HDL-chol respectively [19]. A correlation between Apo B and total cholesterol, LDL-cholesterol and triglycerides was also observed in patients with a recent history of MI [20]. Since LDL-cholesterol has been used to assess the risk associated with CVD [12], coupled with the fact that LDL-cholesterol is positively correlated with Apo B, the latter is expected to be associated with an increased risk of CVDs. Apo B demonstrates its role as a marker of increased CVD risk, together with other biomarkers reflecting lipid-associated risk, either already established, such as cholesterol, or recently investigated, such as lipoprotein(a) [21].

### 3.2. Apolipoprotein B and Carotid Plaques

The association between atherosclerosis markers and Apo B was also investigated in our study. We found that high levels of Apo B correlate with the presence of significant carotid plaques. These data suggest a predictive role of Apo B for carotid atherosclerotic disease even from an asymptomatic stage with, moreover, the potential to be used in screening for carotid disease. However, the results in the literature regarding the association between Apo B and carotid atherosclerosis are controversial.

The analysis of a subcohort of the MultiEthnic Study of Atherosclerosis revealed an association between Apo B and the presence of atherosclerotic plaques at the carotid level. Univariate regression highlighted the fact that Apo B is one of the best predictors of the progression of carotid plaques, with a rate of 13.1 μm per year (*p* = 0.001) [22]. The level of Apo B was significantly associated with increased carotid intima-media thickness in a population of over 6000 patients. The percentage of patients with elevated carotid intima-media thickness increased from 14.3% in patients with Apo B < 78 mg/dL to 22.2% in patients with Apo B > 108 mg/dL [23]. In a population of patients who underwent peritoneal dialysis between 2011 and 2018, it was observed that patients with carotid plaques presented significantly higher values of Apo B than those without plaques at the carotid level (66 ± 17 mg/dL vs. 72 ± 17 mg/dL; *p* = 0.01) [24].

On the other hand, in another study, conducted with pediatric patients (aged between 8 and 17 years), Apo B was not associated with changes in carotid intima-media thickness, but a correlation was observed with levels of arterial stiffness [25]. In a study conducted with 90 patients with ischemic stroke, no significant difference was observed between the levels of Apo B in patients with carotid stenoses versus those without carotid stenoses (*p* = 0.2) [26].

### 3.3. Apolipoprotein B and Serum Levels of Uric Acid

Uric acid is a known marker of CVD and has been associated with different complications of atherosclerosis, such as ventricular arrhythmia and acute coronary syndromes [27,28]. Moreover, data in the literature indicate a strong association between uric acid value and atherosclerosis. The results of a meta-analysis showed a positive relationship between the level of uric acid and subclinical atherosclerosis, quantified by a carotid intima-media thickness index [29]. In the present study, we found that increased levels of uric acid are associated with increased Apo B, similar to the findings of the Brisighella Heart Study [30]. In the NHANES III population, a significant correlation was observed between Apo B and uric acid levels, a correlation that persists even after adjusting for possible confounding factors [31]. In a study published in 2014, low Apo B levels were associated with asymptomatic hyperuricemia, while increased Apo B values were associated with gout [32]. The correlation between serum uric acid and Apo B suggests that the latter can be used as a marker of CVD.

Inflammation is a key player in the complex process of atherosclerosis progression, starting from the early stages of atheroma initiation to the postinfarction phase [33]. Since uric acid is a well-known inflammatory marker [34], Apo B may predict the inflammation underlying different CVDs, such as atherosclerotic disease. In addition, hyperuricemia is involved in the initiation and progression of atherosclerosis through oxidative stress, the latter contributing to the pathogenesis of atherosclerosis via xanthine oxidase, an enzyme that generates reactive oxygen species. By being essential in the production of uric acid, xanthine oxidase represents a link between hyperuricemia and atherosclerosis [35]. Although the available data are quite limited, it seems that the center of the Apo B–uric acid—atherosclerosis triangle is represented by xanthine oxidase. An experimental study published in 2020 showed that the inhibition of xanthine oxidase has anti-LDL oxidation effects, which suggests a possible effect in reducing the progression of atherosclerosis [36].

### 3.4. Apolipoprotein B, Fasting Glucose Level and Diabetes Mellitus

Among women, but not men, without a history of diabetes, Apo B was significantly associated with the value of glycosylated hemoglobin over a 2-year follow-up period [37]. In an Asian population, Apo B, but not LDL-cholesterol, was positively associated with glycosylated hemoglobin [38]. A study from China, carried out with more than 1000 patients, showed an association of Apo B with fasting glucose and type 2 diabetes mellitus, suggesting a predictive role of Apo B for diabetes [39]. Over a 5-year follow-up period, elevated Apo B levels were associated with a 62% risk of developing diabetes mellitus, independent of other variables [40]. Using multivariable Mendelian randomization methods, data from a UK Biobank study shows that Apo B increases the risk of type 2 diabetes mellitus, while LDL-cholesterol seems to decrease the risk of diabetes [41].

In our study, an association was found between Apo B levels and fasting blood glucose values, and between Apo B and glycosylated hemoglobin, but no association between Apo B and the diagnosis of diabetes mellitus was observed. This might suggest that Apo B could be a marker of an increased risk of diabetes mellitus development or a marker of metabolic imbalance rather than a marker of diabetes mellitus per se.

## 4. Materials and Methods

### 4.1. Study Population

This study was conducted with a representative sample of the adult population in Romania arising from the Epidemiological Study on the Prevalence of Arterial Hypertension and Cardiovascular Risk in Romania (SEPHAR) IV. The selection methodology of patients was previously described in detail [42,43]. A total of 811 adult patients under the age of 80 from the general population were included from 10 study centers across Romania (Bucharest, Arad, Cluj-Napoca, Constanța, Craiova, Iași, Oradea, Pitești, Târgu-Mureș and the urban area of Timișoara and its surrounding rural locality) between May and July 2021.

### 4.2. Study Groups

In all patients, the serum level of Apo B was determined in mg/dL using nephelometry (BN ProSpec, Siemens). Based on serum Apo B levels, the patients were divided into two groups: group 1 with low Apo B levels below 130 mg/dL (*n* = 713) and group 2 with high Apo B levels above 130 mg/dL (*n* = 98). Subsequently, the study population was divided into four risk subgroups according to the Apo B cut-offs recommended by the 2021 ESC Guidelines on cardiovascular disease prevention in clinical practice: subgroup A with Apo B < 65 mg/dL (*n* = 102), subgroup B with Apo B between 65 and 80 mg/dL (*n* = 180), subgroup C with Apo B between 81 and 100 mg/dL (*n* = 221) and subgroup D with Apo B > 100 mg/dL (*n* = 308) [16].

### 4.3. Collected Data

Personal and family history of premature CVD (defined as an event at ≤55 years for men or ≤60 for women) [16] and demographic data as well as atherosclerosis risk factors (smoking, obesity, hypertension and diabetes mellitus) were assessed. History of heart failure (HF), myocardial infarction (MI) or angina pectoris, aorto-coronary bypass (CABG) or percutaneous coronary intervention (PCI), atrial fibrillation, stroke and chronic kidney disease (CKD) were obtained from anamneses or previous medical documents. Ankle brachial index was determined using an automatic system and peripheral arterial disease was defined as an index value below 0.9 in any of the two inferior limbs. For the evaluation of carotid atherosclerotic plaques, bilateral B-mode carotid ultrasound was performed for each patient. Image acquisition was performed using an ultrasound. Images of the common carotid artery, carotid bulb and the internal and external carotid arteries were analyzed separately by two experienced persons for the identification of significant atherosclerotic plaques. A carotid plaque was defined as a focal wall thickening of at least 1.5 cm or a focal spot thickening of over 50% relative to the surrounding intima-media thickness. Biochemistry analyzes (lipid levels, K+, uric acid, creatinine, fasting glucose and glycosylated hemoglobin) were obtained for all patients.

### 4.4. Statistical Analysis

Statistical analysis was performed using GraphPad InStat 3.10 software (GraphPad Software Inc., San Diego, CA, USA). Normality tests were performed for all data prior to statistical analysis. Between-group comparisons were performed using unpaired Student’s *t*-tests or Mann–Whitney U-tests for numerical data and chi-squared tests for categorical data. Multiple comparisons were performed using ANOVA tests to establish coefficients of variation, and a comparison of the homogeneity of the coefficients of variation was performed for each variable. Univariate regression analysis was performed to study the association between Apo B levels and biochemistry parameters. In order to test the capacity of Apo B to predict the presence of carotid plaques, we used receiver operating characteristic (ROC) analysis. The results are reported as numbers and percentages for categorical data and as mean ± standard deviation (SD) for numerical variables. The α value was set at 0.05 for statistical significance.

## 5. Conclusions

The results of this analysis of the nationwide registry on hypertension in Romania indicate that high Apo B may be considered as a risk factor for CVD, promoting atherosclerosis and associated with increased expression of classical markers of clinical or subclinical CVD. Elevated levels of Apo B are associated with an altered lipid profile, poorer glycemic control, significant carotid plaques and higher inflammatory status expressed by increased uric acid. These findings suggest that elevated Apo B is a risk factor for CVDs and is involved in different stages of atherosclerosis progression.

## Figures and Tables

**Table 1:** Atherosclerosis risk factors and comorbidities in the low versus high Apo B groups.

	Group 1 (<130 mg/dL)	Group 2 (>130 mg/dL)	*p*-Value
Active smoking	614 (86%)	75 (77%)	**0.01**
Obesity	313 (44%)	53 (54%)	0.06
Hypertension	259 (36%)	43 (44%)	0.1
Diabetes mellitus	71 (10%)	12 (12%)	0.4
HF	45 (6%)	2 (2%)	0.1
MI	16 (2%)	5 (5%)	0.4
Angina pectoris	64 (9%)	13 (13%)	0.1
PCI or CABG	28 (3%)	1 (1%)	0.2
Atrial fibrillation	52 (7%)	5 (5%)	0.5
Stroke	27 (4%)	7 (7%)	0.1
Peripheral arterial disease	15 (2%)	4 (4%)	0.2
CKD	10 (1%)	3 (3%)	0.2

The values are expressed as absolute values and percentages; *p*-values refer to between-group comparisons based on a chi-squared test. HF—heart failure; MI—myocardial infarction; PCI—percutaneous coronary intervention; CABG—aortocoronary bypass; CKD—chronic kidney disease.

**Table 2:** Lipid profile in low versus high Apo B groups.

Parameter	Group 1 (<130 mg/dL)	Group 2 (>130 mg/dL)	*p*-Value
Total cholesterol (mg/dL)	192.0 ± 36.3 [189.6–194.5]	289.0 ± 48.7 [270.3–289.8]	<0.0001
Triglycerides (mg/dL)	111.4 ± 67.1 [106.6–116.2]	201.9 ± 120.1 [176.4–227.4]	<0.0001
HDL-cholesterol (mg/dL)	53.9 ± 13.7 [53.0–54.9]	50.2 ± 12.5 [47.7–52.7]	0.005
LDL-cholesterol (mg/dL)	123.4 ± 35.0 [121.0–125.8]	201.5 ± 43.4 [192.8–210.2]	<0.0001

Values are expressed as mean ± standard deviation [95% confidence interval]; *p*-values refer to between-group comparisons based on an unpaired Student’s *t*-test. HDL—high-density lipoprotein; LDL—low-density lipoprotein.

**Table 3:** Lipid profile in the Apo B risk subgroups.

Parameter	Apo B < 65 mg/dL *n* = 102	Apo B 65–80 mg/dL *n* = 180	Apo B 81–100 mg/dL *n* = 221	Apo B > 100 mg/dL *n* = 308	*p*-Value ANOVA	*p*-Value of CV
Total cholesterol (mg/dL)	144.0 ± 20.3 (14%) [140.1–147.8]	171.1 ± 21.0 (12%) [167.6–174.5]	195.3 ± 23.2 (12%) [192.5–198.1]	239.8 ± 42.0 (18%) [235.4–244.1]	<0.0001	<0.0001
Triglycerides (mg/dL)	71.8 ± 42.2 (59%) [63.3–80.4]	95.1 ± 56.6 (60%) [86.8–103.5]	106.2 ± 58.6 (55%) [98.8–149.8]	159.9 ± 93.3 (58%) [149.8–170.0]	<0.0001	0.85
HDL-cholesterol (mg/dL)	57.9 ± 13.9 (24%) [55.3-60.5]	56.5 ± 14.4 (25%) [54.4–58.5]	53.7 ± 14.2 (26%) [52.0–55.4]	50.5 ± 12.0 (24%) [49.3–51.8]	<0.0001	0.38
LDL-cholesterol (mg/dL)	73.4 ± 15.5 (21%) [70.5–76.3]	101.7 ± 21.8 (21%) [98.6–104.8]	127.5 ± 19.6 (15%) [125.1–129.8]	168 ± 36.4 (22%) [164.9–172.5]	<0.0001	<0.0001

The values are expressed as mean ± standard deviation (coefficient of variation %) [95% confidence interval]; *p*-values refer to between-group comparisons based on an ANOVA test. CV—coefficient of variation; HDL—high-density lipoprotein; LDL—low-density lipoprotein.

**Table 4:** Biochemistry parameters in the Apo B risk subgroups.

Parameter	Apo B < 65 mg/dL *n* = 102	Apo B 65–80 mg/dL *n* = 180	Apo B 81–100 mg/dL *n* = 221	Apo B > 100 mg/dL *n* = 308	*p*-Value	*p*-Value of CV
Uric acid (mg/dL)	4.8 ± 1.3 (27%) [4.4–1.3]	5.0 ± 1.6 (32%) [4.6–5.3]	5.1 ± 1.5 (29%) [4.8–5.4]	5.8 ± 1.6 (28%) [5.5–6.1]	<0.0001	0.15
Fasting glucose (mg/dl)	96.9 ± 24.8 (26%) [91.8–102.0]	98.6 ± 24.4 (25%) [95.1–102.2]	96.8 ± 16.6 (17%) [94.7–98.8]	104.4 ± 25.8 (25%) [101.7–107.2]	<0.0001	<0.0001
HbA1c%	5.5 ± 0.9 (16%) [5.3–5.6]	5.5 ± 0.7 (13%) [5.4–5.6]	5.5 ± 0.5 (9%) [5.4–5.6]	5.8 ± 0.9 (16%) [5.7–5.9]	<0.0001	<0.0001

The values are expressed as mean ± standard deviation (coefficient of variation %) [95% confidence interval]; *p*-values refer to between-group comparisons based on an ANOVA test. CV—coefficient of variation; HbA1c—glycosylated hemoglobin.

## Data Availability

Archived datasets are available upon request by any interested third party.
